# Response to cabergoline treatment, gonadal axis recovery, and outcomes of drug withdrawal, in men with microprolactinoma: a retrospective cohort study

**DOI:** 10.1007/s12020-025-04215-w

**Published:** 2025-04-09

**Authors:** Yaron Rudman, Neta Simon, Rona Shimon, Genady Drozdinsky, Efrat Markus, Hadar Duskin-Bitan, Hiba Masri-Iraqi, Gloria Tsvetov, Amit Akirov, Ilan Shimon

**Affiliations:** 1 Institute of Endocrinology, Beilinson Hospital, Rabin Medical Center, Petah Tikva, Israel; 2 Faculty of Medical and Health Sciences, Tel Aviv University, Tel Aviv, Israel; 3 Faculty of Medicine, Semmelweis University, Budapest, Hungary; 4 Infectious Diseases Unit, Rabin Medical Center, Petah Tikva, Israel; 5 Institute of Endocrinology, Assaf Harofeh Medical Center, Zerifin, Beer Ya’akov, Israel

**Keywords:** Prolactin, Prolactinoma, Pituitary microadenoma, Cabergoline, Men

## Abstract

**Purpose:**

Due to the low incidence of male microprolactinoma, there is a paucity of data in the literature regarding its management. Our aim was to investigate the long-term outcomes of cabergoline treatment in men with microprolactinoma.

**Methods:**

In this single-center retrospective cohort study, we reviewed patient’s records at prolactinoma diagnosis, following cabergoline discontinuation (if occurred), and at the last clinic visit. We collected all available clinical data, laboratory tests, and pituitary magnetic resonance imaging. We report response rates, gonadal axis recovery, and outcomes following cabergoline discontinuation.

**Results:**

The study cohort included 47 men with microprolactinoma [age at diagnosis 45.6 ± 17.6 years; median prolactin 70.0 ng/ml (IQR 51.0–103.4); low testosterone, 34 men (72.3%); adenoma diameter 5.6 ± 2.0 mm; median follow-up 7.1 years (IQR 3.5–10.4)]. Forty-two patients (89.4%) achieved normal prolactin levels within a median treatment time of 4.0 months (IQR 3.0–5.5) and had normal testosterone at last clinic visit. Five men (10.6%) did not achieve prolactin normalization, of whom 3 men remained hypogonadal. Mild side effects occurred in 4.3% of patients and disappeared with dose reduction. Thirteen men that achieved normal prolactin attempted drug discontinuation, but only 5 remained with normoprolactinemia. Men who maintained normal prolactin levels were treated longer with cabergoline [median treatment of 10 years (IQR 4.6–10.3) vs 2.0 years (IQR 1.5–3.2); *p* < 0.01].

**Conclusions:**

Ninety percent of men harboring microprolactinoma achieved normoprolactinemia and subsequent testosterone normalization with cabergoline treatment. Men that discontinued cabergoline after prolonged prolactin suppression (>5 years) achieved sustained remission. These findings assist informed decision-making, between medical and surgical treatment.

## Introduction

Prolactin-secreting adenomas (i.e., prolactinomas) are the most common type of functional pituitary tumors, accounting for approximately 40 percent of all pituitary tumors [1–3]. Prolactinomas are classified according to their size: microprolactinomas are smaller than 10 mm in diameter, whereas macroprolactinomas are ≥10 mm. While macroadenomas tend to extend beyond the sella turcica and cause a variety of symptoms resulting from the pressure exerted on adjacent tissues (e.g., visual field defect, hypopituitarism), microprolactinomas are small and rarely protrude outside the pituitary gland and thus their effect is usually limited to increased prolactin secretion [4, 5]. Importantly, microprolactinoma represents a separate diagnostic entity, as progression from microadenoma to macroadenoma is rare [6].

The prevalence of clinically relevant microprolactinomas varies greatly according to gender: over 70 percent of prolactinomas are microadenomas in women, compared to less than 25 percent in men [7]. As the incidence of prolactinoma in the general population is not high, estimated at 40 cases per 1,000,000 per year [8], with only 20 percent of prolactinomas occur in men [3, 9] - male microprolactinoma is a rare medical condition, with an estimated incidence of less than 2 cases per 1,000,000 per year. However, according to autopsy studies, most prolactinomas are microprolactinomas that do not cause symptoms and their prevalence is much higher than those that are clinically apparent [10]. Thus, symptomatic microprolactinomas probably represent only “the tip of the iceberg”.

Male patients presenting with microprolactinomas suffer from hyperprolactinemia and secondary hypogonadism, and commonly seek medical attention due to sexual dysfunction (erectile dysfunction and/or low libido), gynecomastia, or rarely galactorrhea [11]. The goals of treatment in male microprolactinoma include serum prolactin normalization, and resolution of hypogonadism and sexual dysfunction with improvement in libido, and restoration of fertility [12]. Drug treatment with cabergoline, a potent dopamine agonist with long-acting effect, is the preferred first line of treatment for prolactinomas [13]. However, the use of surgery as primary treatment for microprolactinomas in those who are suitable for this approach is becoming more popular, obviating the need for continuous drug therapy [14, 15]. Additionally, in some cases where symptoms are very mild or absent patients can simply be monitored without treatment [11, 12].

Previous studies investigating drug treatment outcomes included very small samples of men with microprolactinoma [4, 5, 16, 17], with the largest series including 15 men. Notably, under certain conditions, cabergoline for the treatment of prolactinomas can be safely discontinued, particularly after sustained normalization of prolactin [18]. However, data on discontinuation in men with microprolactinoma are lacking, as the largest study examining cabergoline withdrawal included 11 men with microprolactinoma [19].

Considering the paucity of data currently available in the literature, and given the alternative of pituitary surgery, we see great importance in better understanding the outcomes of drug treatment in this unique patient population. This single-center retrospective study reports the long-term follow-up of 47 men with microprolactinoma treated with cabergoline. We aimed to examine the response rates to cabergoline therapy, the gonadal axis recovery rates, and the outcomes of cabergoline withdrawal.

## Materials and methods

### Patients

The study population included adult men (over 18 years of age) with pituitary microadenomas (smaller than 10 mm in diameter) and persistently elevated prolactin levels, with serum prolactin ≥ 30 ng/ml on at least 2 separate measurements. An additional mandatory inclusion criterion was the presence of signs and symptoms of hyperprolactinemic hypogonadism (decreased libido, erectile dysfunction, gynecomastia, galactorrhea), or laboratory findings consistent with central hypogonadism. Secondary causes of hyperprolactinemia were excluded: treatment with drugs that cause hyperprolactinemia, hypothyroidism, and liver or kidney disease. Macroprolactin was tested in all cases where a patient was asymptomatic and had normal or borderline total testosterone levels.

Patients were identified by reviewing our single center prolactinoma registry. All patients were either diagnosed or referred immediately after diagnosis to the Pituitary Clinic at Rabin Medical Center, Israel. All patients were treated with cabergoline and were followed by the clinic’s endocrinologists.

Patients who underwent pituitary surgery and those who were followed without any treatment were excluded. Patients who were concomitantly treated with antipsychotic medications were excluded. Patients with insulin-like growth factor-1 (IGF-1) above 1.3 times the upper limit of normal or those with clinical acromegaly were also excluded.

The study was approved by the Rabin Medical Center institutional review board, with waiver of patient consent, and was performed in line with the principles of the Declaration of Helsinki. The authors received no funding for this study.

### Data collection

The collected Information included: clinical presentation and response to therapy, laboratory tests at presentation and during follow-up, including prolactin, total testosterone, luteinizing hormone (LH), follicle-stimulating hormone (FSH), IGF-1, morning cortisol, thyroid-stimulating hormone (TSH) and free levothyroxine (FT_4_) measurements. Hemoglobin levels were also recorded, as anemia is a marker of hyperprolactinemia in male prolactinoma [20]. Macroprolactin was not routinely assessed.

Patients underwent pituitary-focused magnetic resonance imaging (MRI) at baseline. Patients were included in the study only if a pituitary microadenoma was reported in the original radiological report, interpreted by an expert neuroradiologist. In most cases, an additional pituitary MRI was performed within 12 months after prolactinoma diagnosis. Yet, as the current consensus guidelines do not require repeat MRI in cases of prolactin normalization in response to cabergoline treatment [15], some of the recently diagnosed patients did not have imaging follow-up.

All data were collected at 4 time points: at prolactinoma diagnosis (baseline), 12 months after cabergoline treatment initiation, at the time of drug discontinuation (if occurred), and at the last clinic visit (i.e., at the end of follow-up).

Data regarding the timing of cabergoline treatment initiation, prolactin normalization, testosterone normalization, testosterone replacement initiation (when given), and cabergoline discontinuation (if occurred) were collected.

### Treatment protocol

Medical treatment with cabergoline was initiated at a weekly dose of 0.5 mg, with dose adjustments every 3–6 months according to prolactin levels, as needed, until prolactin reached either normalization or plateau. No predetermined limit was set for the maximal cabergoline dose. After 12–24 months of cabergoline treatment, dose reduction was considered to allow the minimal dose needed to control prolactin. Treatment withdrawal was performed after at least 24 months of medical treatment, in patients whose prolactin levels had normalized with low doses of cabergoline (i.e., 0.25 mg/week). The timing of drug discontinuation was not determined according to a uniform protocol but rather on a case-by-case basis, at the discretion of the treating physician. Treatment discontinuations that resulted from the patient’s decision and did not meet the above criteria did occur and were documented as well. Patients that suffered from central hypogonadism persistence, that is low total testosterone and symptoms of hypogonadism despite ≥6 months of normal prolactin levels, were offered testosterone therapy.

In the event of testosterone replacement, the last testosterone, LH and FSH measurements recorded just before replacement treatment was initiated were documented as end of follow-up measurements.

### Biochemical evaluation

Serum prolactin levels were measured by immunometric assay (Immulite 2000; Siemens), which has a sensitivity of 0.15 ng/ml. The intra-assay coefficients of variation (CVs) for prolactin concentrations of 22 and 164 ng/ml were 2.3% and 3.8%, respectively; the corresponding inter-assay CV was 6%. Serum prolactin reference levels for men in our laboratory are 2–20 ng/ml. Reference levels for testosterone in men in our laboratory are 2.8–9.6 ng/ml. Central hypogonadism was defined as low serum total testosterone (< 2.8 ng/ml) with low or inappropriately normal LH levels. Secondary adrenal insufficiency was defined as 9:00 a.m. cortisol value below 100 nmol/L or below 450 nmol/L following adrenocorticotropic hormone (ACTH) stimulation. Central hypothyroidism was defined as low or inappropriately normal TSH levels in the presence of low FT_4_ levels. We defined anemia in adult men as hemoglobin concentration of 13.5 g/dL or less, according to the definition of the American Society of Hematology [21]. The reference levels for the laboratory tests were determined according to the manufacturer’s instructions.

### Statistical analysis and plan

Statistical analysis was performed using IBM SPSS version 22.0 (IBM Corp., Armonk, NY). We described the baseline characteristics of the entire cohort. We compared the baseline characteristics and response to medical treatment after 12 months of cabergoline treatment and at last clinic visit between patients who achieved prolactin normalization (responders) and those who did not achieve normal prolactin levels (non-responders). The follow-up time began at prolactinoma diagnosis and ended at the last clinic visit or at the event of drug discontinuation or testosterone replacement. Finally, we compared patients’ characteristics and outcomes of cabergoline discontinuation between patients without recurrence of hyperprolactinemia and those who experienced hyperprolactinemia recurrence. Follow-up for this analysis began at the time of drug discontinuation and ended at the last clinic visit or in the event of prolactin increase above the upper limit of the normal range. Continuous variables were presented by Mean (SD) or Median (IQR) as appropriate. Dichotomous variables were presented by N (%). The T-test and Mann–Whitney tests were used to compare the values of normally and non-normally distributed continuous variables among the two groups, with Chi-Square test used for comparison of categorical variables. Two-sided P-values less than 0.05 were considered statistically significant.

## Results

**Fig. 1:**
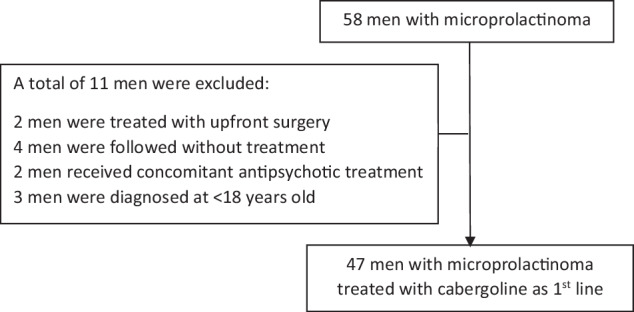
Patient selection flowchart

From January 2004 to December 2024, a total of 58 male patients with microprolactinoma were identified. After careful reviewing each case, 11 patients were excluded. Excluded were 2 patients who had undergone upfront pituitary surgery due to patient preference, 2 elderly men with mildly symptomatic central hypogonadism who chose not to receive treatment at all, and 2 asymptomatic men with borderline total testosterone that were diagnosed after the treating physician ruled out secondary causes of hyperprolactinemia (including macroprolactin, medications, hypothyroidism, and liver or kidney disease). The reasons for exclusion of all patients are detailed in Fig. 1.

A total of 47 cabergoline-treated men with microprolactinoma were included in the final cohort (Table 1). The reasons for the initial clinic referral for all included patients are shown in Table 2.

**Table 1:** Baseline characteristics of 47 male patients with microprolactinoma

Variable	Male microprolactinoma cohort (*N* = 47)
Age at diagnosis, years - mean (SD)	45.6 (17.6)
Prolactin, ng/ml - median (IQR)	70.0 (51.0–103.4)
Low testosterone - n (%)	34 (72.3%)
Testosterone, ng/ml - mean (SD)	2.7 (1.7)
Luteinizing hormone, mIU/ml - mean (SD) ^a^	2.7 (1.6)
Follicle-stimulating hormone, mIU/ml - mean (SD) ^a^	5.3 (4.2)
Adenoma maximal diameter, mm - mean (SD)	5.6 (2.0)
Cavernous sinus invasion - n (%)	0 (0.0%)
Sphenoid sinus invasion - n (%)	1 (2.1%)
Suprasellar extension - n (%)	2 (4.3%)
Central hypothyroidism - n (%)	0 (0.0%)
Central adrenal insufficiency - n (%)	0 (0.0%)
Visual field defect - n (%)	0 (0.0%)
Sexual dysfunction - n (%) ^b^	36 (78.3%)
Headache – n (%) ^c^	6 (14.2%)
Hemoglobin, g/dL - mean (SD) ^d^	13.7 (0.8)
Anemia - n (%) ^d^	23 (50.0%)
Follow-up, years - median (IQR)	7.1 (3.5–10.4)

^a^ *n* = 33

^b^ *n* = 46

^c^ *n* = 42

^d^ *n* = 46

**Table 2:** Reasons for initial clinic referral

Reasons for initial clinic referral	*n* (%)
Sexual dysfunction	29 (61.8%)
Gynecomastia	7 (14.9%)
Sexual dysfunction & Gynecomastia	3 (6.4%)
Galactorrhea	3 (6.4%)
Infertility	2 (4.2%)
Low bone mineral density	2 (4.2%)
Headaches	1 (2.1%)
**Total**	**47** (**100.0%)**

### Baseline characteristics of male microprolactinoma

For the entire cohort (*N* = 47), the mean age at diagnosis was 45.6 ± 17.6 years, with median prolactin levels of 70.0 ng/ml (IQR 51.0–103.4), and a mean adenoma diameter of 5.6 ± 2.0 mm (Table 1). The median follow-up was 7.1 years (IQR 3.5–10.4). Low testosterone levels were evident in 34 men (72.3%), while complaints of sexual dysfunction were recorded in 78.3% of patients. Anemia was present in 23 out of 46 patients (50.0%). All baseline patient characteristics are elaborated in Table 1.

The median cabergoline dose in the entire cohort was 0.5 mg/week (IQR 0.5–1.0). Side effects of cabergoline were reported in 2 men (4.3%), both received 0.5 mg/week: one patient reported hypersexuality, and another patient reported mild treatment-related headaches. In both cases, the side effects resolved after reducing the cabergoline dose to 0.25 mg/week, without a concomitant increase in prolactin levels.

### Response to cabergoline treatment

All 47 men were treated with cabergoline, with a minimum follow-up time of 18 months.

We divided our cohort into two groups: men who achieved normal prolactin levels with cabergoline treatment (responders), and men who did not (non responders). A total of 42 patients (89.4%) achieved normal prolactin levels, within a median treatment time of 4.0 months (IQR 3.0–5.5). Of note, in 3 cases the time to prolactin normalization was longer than 12 months. However, 5 men (10.6%) did not achieve prolactin normalization at the end of follow-up. Patients who did not achieve prolactin normalization were treated with higher doses of cabergoline (median 1.5 mg/week, IQR 1.5–2.5), compared to men who normalized prolactin (median dose, 0.5 mg/week, IQR 0.5–0.5; *p* < 0.01). There were no differences in baseline characteristics between patients who responded to cabergoline treatment (*N* = 42) and those who did not respond (*N* = 5), as shown in Table 3. All patients who achieved normal prolactin levels with cabergoline showed normal gonadal axis function at the end of follow-up, compared with 2 patients (40.0%) whose testosterone was normal at the end of follow-up despite high prolactin levels. Of these 2 men, one patient had normal testosterone levels already at diagnosis, whereas another patient achieved normal testosterone after 10 years of cabergoline treatment (yet, despite below-normal testosterone levels, the patient achieved significant improvement in sexual function early in the treatment). In patients who responded to cabergoline, the median time from prolactin normalization to recovery of eugonadism was 2.0 months (IQR 0.8–7.2). Improvement of sexual function was noted in 23 out of 28 men (82.1%) who normalized prolactin, and in 1 out of 4 men (25.0%) who did not (*p* = 0.03; Table 3).

**Table 3:** Baseline characteristics and response to medical treatment after 12 months of cabergoline treatment and at last clinic visit in patients who achieved prolactin normalization (*N* = 42) and in patients who did not achieve prolactin normalization (*N* = 5)

	Patients who achieved prolactin normalization (*N* = 42)	patients who did not achieve prolactin normalization (*N* = 5)	*p*-value
**Baseline / At diagnosis**			
Age at diagnosis, years - mean (SD)	45.3 (17.8)	47.8 (18.1)	0.77
Prolactin, ng/ml - median (IQR)	70.0 (50.8–102.4)	102.0 (51.7–309.5)	0.25
Low testosterone - n (%)	30 (73.2%)	4 (80.0%)	0.74
Testosterone, ng/ml - mean (SD)	2.7 (1.8)	2.6 (0.9)	0.90
Luteinizing hormone, mIU/ml - mean (SD) ^a^	2.7 (1.7)	2.8 (0.7)	0.88
Follicle-stimulating hormone, mIU/ml - mean (SD) ^a^	5.2 (4.4)	6.5 (3.8)	0.54
Adenoma maximal diameter, mm - mean (SD)	5.4 (2.0)	6.8 (1.8)	0.15
Cavernous sinus invasion - n (%)	0 (0.0%)	0 (0.0%)	–
Sphenoid sinus invasion - n (%)	1 (2.4%)	0 (0.0%)	–
Suprasellar invasion - n (%)	2 (4.8%)	0 (0.0%)	–
Central hypothyroidism - n (%)	0 (0.0%)	0 (0.0%)	–
Central adrenal insufficiency - n (%)	0 (0.0%)	0 (0.0%)	–
Visual field defect - n (%)	0 (0.0%)	0 (0.0%)	–
Sexual dysfunction - n (%) ^b^	31 (75.6%)	5 (100.0%)	0.57
Headache – n (%) ^c^	4 (10.8%)	2 (40.0%)	0.15
Hemoglobin, g/dL - mean (SD) ^d^	13.6 (0.8)	14.0 (0.6)	0.29
Anemia - n (%) ^d^	22 (53.7%)	1 (20.0%)	0.16
**After 12 months of cabergoline treatment**			
Prolactin, ng/ml - median (IQR)	9.0 (5.3–13.0)	26.0 (24.0–98.0)	<0.01
Hyperprolactinemia - n (%)	3 (7.1%)	5 (100.0%)	<0.01
Low testosterone - n (%) ^e^	7 (17.1%)	4 (80.0%)	<0.01
Testosterone, ng/ml - mean (SD)	4.7 (1.9)	2.8 (0.5)	0.03
Adenoma maximal diameter, mm - mean (SD) ^f^	4.0 (2.1)	6.0 (2.2)	0.06
No adenoma shrinkage - n (%) ^f^	14 (38.9%)	2 (40.0%)	0.96
**Last clinic visit / At the end of follow-up**			
Prolactin, ng/ml - median (IQR)	7.0 (3.4–10.9)	29.8 (27.0–85.0)	<0.01
Hyperprolactinemia - n (%)	0 (0.0%)	5 (100.0%)	<0.01
Low testosterone - n (%)	0 (0.0%)	3 (60.0%)	<0.01
Testosterone, ng/ml - mean (SD)	5.4 (1.9)	3.4 (1.2)	0.02
Luteinizing hormone, mIU/ml - mean (SD) ^a^	4.8 (3.0)	3.9 (1.4)	0.50
Follicle-stimulating hormone, mIU/ml - mean (SD) ^a^	8.1 (4.8)	8.3 (6.2)	0.93
Adenoma maximal diameter, mm - mean (SD) ^f^	2.3 (2.1)	4.1 (3.5)	0.10
No adenoma shrinkage - n (%) ^f^	6 (16.7%)	1 (20.0%)	0.85
Sexual function improvement – n (%) ^g^	23 (82.1%)	1 (25.0%)	0.03
Hemoglobin, g/dL - mean (SD) ^d^	14.5 (0.9)	14.3 (1.0)	0.63
Anemia - n (%) ^d^	5 (12.2%)	1 (20.0%)	0.62
Maximal Cabergoline dose, mg/week – median (IQR)	0.5 (0.5–0.5)	1.5 (1.5–2.5)	<0.01
Follow-up, years - median (IQR)	6.8 (2.8–10.1)	8.0 (3.8–14.4)	0.43

^a^ *n* = 28/42 and *n* = 5/5

^b^ *n* = 41/42 and 5/5

^c^ *n* = 37/41 and *n* = 5/5

^d^ *n* = 41/42 and *n* = 5/5

^e^ *n* = 41/42 and *n* = 5/5

^f^ *n* = 36/42 and *n* = 5/5

^g^ *n* = 28/42 and 4/5

**Fig. 2:**
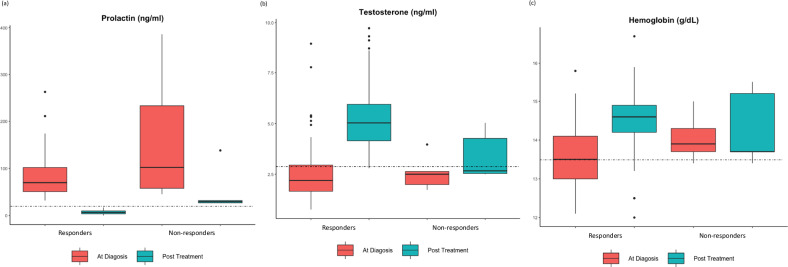
Box plot diagrams showing (**a**) prolactin, (**b**) testosterone, and (**c**) hemoglobin levels at the time of prolactinoma diagnosis and at the end of follow-up (following cabergoline treatment), in 42 men with microprolactinoma who achieved normal prolactin levels (responders) compared with 5 men with microprolactinoma who did not achieve normal prolactin levels (non-responders)

In patients who responded to treatment (*N* = 42), prolactin dropped from a median of 70.0 ng/ml to 7.0 ng/ml (*p* < 0.01), testosterone increased from 2.7 ± 1.8 ng/ml to 5.4 ± 1.9 ng/ml (*p* < 0.01), and the average hemoglobin level increased from 13.6 ± 0.8 g/dL at diagnosis to 14.5 ± 0.9 g/dL (*p* < 0.01; Fig. 2). In those who did not respond to cabergoline (*N* = 5), the median prolactin was 102 ng/ml at diagnosis and 29.8 ng/ml at the end of follow-up (*p* < 0.01), testosterone was 2.6 ± 0.9 ng/ml at baseline and 3.4 ± 1.2 ng/ml at the last clinic visit (*p* = 0.34), and hemoglobin was 14.0 ± 0.6 g/dL and 14.3 ± 1.0 g/dL (*p* = 0.02), respectively (Fig. 2). Of the 5 patients who did not respond to treatment with cabergoline, 3 patients remained hypogonadal: a 69-year-old man who complained of erectile dysfunction but ruled out other hypogonadism-related problems (fatigue, depression, etc.) and refused testosterone treatment, a 51-year-old man whose testosterone increased from 1.7 ng/ml to 2.5 ng/ml and his sexual dysfunction improved despite above-normal prolactin levels, and a 57 year-old patient that received testosterone replacement.

### Outcomes of cabergoline discontinuation in 13 men with microprolactinoma

**Fig. 3:**
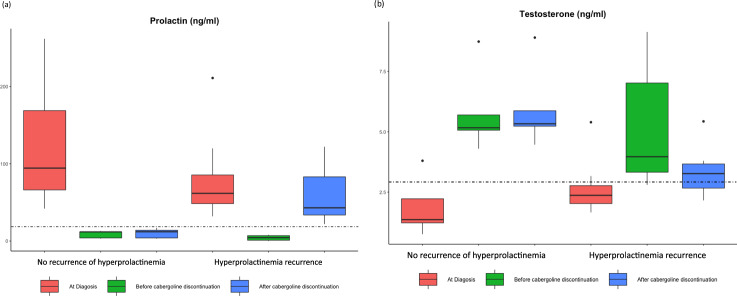
Box plot diagrams showing (**a**) prolactin and (**b**) testosterone levels at the time of prolactinoma diagnosis, before cabergoline discontinuation, and after cabergoline discontinuation, in 5 men with microprolactinoma without hyperprolactinemia recurrence, and in 8 men with recurrent hyperprolactinemia after treatment discontinuation

A total of 13 men achieved prolactin normalization with cabergoline, with subsequent drug discontinuation. Only 5 patients remained in remission et the end of follow-up, while the other 8 exhibited a rise in prolactin levels within 12 months of cabergoline discontinuation (median 6.0 months, IQR 5.0–9.5). The baseline patient characteristics, including patients’ age, prolactin, testosterone, and adenoma diameter are listed in Table 4. At the time of cabergoline discontinuation, both groups had similar prolactin levels [median 6.0 (2.5–10.0) ng/ml vs 4.0 (1.0–7.0) ng/ml], testosterone levels (5.8 ± 1.7 ng/ml vs 5.2 ± 2.4 ng/ml), and mean pituitary adenoma size (3.0 ± 1.4 mm vs 3.8 ± 1.3 mm; Table 4). However, 3 out of 5 men (60%) with sustained remission exhibited no abnormality in their last pituitary MRI, compared to 2 out of 8 men (25.0%) who suffered from hyperprolactinemia recurrence (*p* = 0.22). The weekly cabergoline doses of the two groups were comparable (0.5 ± 0.0 vs. 0.7 ± 0.3 mg/week), yet patients who maintained normal prolactin levels were treated with cabergoline for a significantly longer duration, with a median treatment time of 10 years (IQR 4.6–10.3), compared with a median of 2.0 years (IQR 1.5–3.2) in those with recurrent hyperprolactinemia (*p* < 0.01; Table 4). In the 8 men who experienced recurrence of hyperprolactinemia after discontinuation of cabergoline, prolactin reached a median level of 43 ng/ml (IQR 33.8–83.0), corresponding to 70% of prolactin levels at diagnosis. Similarly, in those with recurrent hyperprolactinemia, testosterone levels decreased from 5.2 ± 2.4 ng/mL at the time of cabergoline discontinuation to 3.4 ± 1.1 ng/mL at the end of follow-up (*p* = 0.10), whereas patients with consistently normal prolactin levels demonstrated stable testosterone levels (Table 4 and Fig. 3).

**Table 4:** Patient characteristics and outcomes of cabergoline discontinuation in 13 men with microprolactinoma: a comparison of patients without recurrence of hyperprolactinemia (*N* = 5) versus those who experienced hyperprolactinemia recurrence (*N* = 8)

	No recurrence of hyperprolactinemia (*N* = 5)	Hyperprolactinemia recurrence (*N* = 8)	p-value
**Baseline / At prolactinoma diagnosis**			
Age at diagnosis, years - mean (SD)	43.8 (13.0)	49.5 (21.9)	0.61
Prolactin, ng/ml - median (IQR)	94.3 (54.0–215.0)	61.0 (41.5–108.5)	0.35
Testosterone, ng/ml - mean (SD)	1.9 (1.2)	2.7 (1.2)	0.27
Adenoma maximal diameter, mm - mean (SD)	6.2 (1.6)	5.0 (2.1)	0.30
**At the time of cabergoline discontinuation**			
Prolactin, ng/ml - median (IQR)	6.0 (2.5–10.0)	4.0 (1.0–7.0)	0.63
Testosterone, ng/ml - mean (SD)	5.8 (1.7)	5.2 (2.4)	0.67
Adenoma diameter (last measurement), mm - mean (SD) ^a^	3.0 (1.4)	3.8 (1.3)	0.18
No pituitary adenoma on last imaging – n (%)	3 (60.%)	2 (25.0%)	0.22
Maximal cabergoline dose, mg/week – mean (SD)	0.5 (0.0)	0.7 (0.3)	0.27
Cabergoline treatment duration, years – median (IQR)	10.0 (4.6–10.3)	2.0 (1.5–3.2)	<0.01
**Outcome of cabergoline discontinuation**			
Duration of cabergoline-free follow-up, months – median (IQR) ^b^	46.0 (33.0–92.0)	6.0 (5.0–9.5)	<0.01
Last prolactin, ng/ml - median (IQR)	12.0 (4.0–14.0)	43.0 (33.8–83.0)	<0.01
Last testosterone, ng/ml - mean (SD)	6.0 (1.7)	3.4 (1.1)	<0.01

^a^ Measured only if adenoma was visible on last pituitary MRI (*n* = 2/5 and *n* = 6/8)

^b^ Follow-up continued until prolactin increased to abnormal levels or until the last clinic visit

## Discussion

The latest consensus on the diagnosis and management of prolactin-secreting pituitary adenomas recommends that pituitary surgery along with dopamine agonist therapy should be considered as a first-line treatment option in patients with microprolactinoma [15]. However, despite the extensive data available on the treatment of prolactinoma in women [22], and recent information on the outcomes of surgical treatment of microprolactinomas [23, 24], there are limited data on the medical treatment of microprolactinoma in men. In this study we retrospectively studied a cohort of 47 men treated with cabergoline, with a median follow-up of 7.1 years (IQR 3.5–10.4), and found that 89.4% of patients achieved normal prolactin levels, after a short time of cabergoline treatment (median 4.0 months, IQR 3.0–5.5). We found that all 42 men that responded to cabergoline had normal testosterone levels at last clinic visit. Among the five men (10.6%) who did not achieve normal prolactin levels, 3 men (60.0%) remained hypogonadal at the end of follow-up.

While previous studies examined outcomes of medical treatment in patients with prolactinomas in a mixed population of both genders [7] and both micro- and macroprolactinomas [4, 5], with very small numbers of male patients with microprolactinomas, our study is dedicated to the investigation of medical therapy outcomes in men with microprolactinomas. In a Belgian series of 455 patients treated with cabergoline for hyperprolactinemia, with 22.0% men and a total of 38.2% microprolactinomas, the response rate to treatment in men with microprolactinomas was 92.0% [7]. In a retrospective study that included 12 men with microprolactinoma (median follow-up of 4.0 years), 10 men (83.3%) responded to treatment with dopamine agonist [4]. An Italian study prospectively evaluated the effect of 24 months of cabergoline therapy in 10 men with microprolactinoma and found a response rate of 80.0% [5]. The median cabergoline dose for male microprolactinoma was 0.5 mg/week in the three studies. In our cohort 10.6% of patients did not achieve prolactin normalization with long-term cabergoline treatment. For comparison, previously reported rates of prolactin normalization in men with macroprolactinomas were slightly worse, ranging from 75.6 to 90 percent [4, 5, 7, 25]. In contrast, prolactin normalization rates in men suffering from symptomatic hyperprolactinemia without a visible pituitary adenoma on imaging have been reported to be 100.0% [26].

At presentation, 72.3% of patients had low testosterone levels, and 78.3% reported sexual dysfunction. Sexual dysfunction was the main reason for initial clinic referral in 32 men (68.1%) and infertility in 2 additional men (4.3%). We found that all 42 patients that responded to cabergoline also normalized their testosterone levels at the end of follow-up, and 23 of 28 men (82.1%) reported improved sexual function. Importantly, the time from prolactin normalization to recovery of eugonadism was relatively short (median 2.0 months, IQR 0.8–7.2). These data highlight the fundamental difference between microprolactinomas and the larger macroprolactinomas, the latter involving a high percentage (20.7–31.0%) of patients with persistent hypogonadism despite normalization of prolactin levels [27–29]. In macroprolactinomas, this permanent gonadal axis damage is mainly attributed to the tumor mass effect.

Anemia accompanies men suffering from hypogonadism and occurs in over 50% of men with macroprolactinoma [20]. In our cohort of male microprolactinoma, anemia was present in 50.0% of patients at the time of diagnosis. Yet, after cabergoline treatment only 13.0% of men suffered from anemia. Following prolactin suppression and testosterone normalization, the average hemoglobin levels rose from 13.6 g/dL at baseline to 14.5 g/dL (Fig. 2). However, in patients who did not achieve normal prolactin, the rise in testosterone was moderate and hemoglobin levels exhibited only a small increase (Table 3 and Fig. 2). The improvement in hemoglobin levels, correlated with the improvement in testosterone levels, is comparable to that seen in men with macroprolactinomas [20].

In the current study cohort, all patients had microprolactinomas without evidence of tumor invasion into the cavernous sinus, per pituitary MRI (Table 1). That is, Knosp classification grade 0 or grade 1 [30], meaning that all included patients were candidates for pituitary surgery [15]. In two recently published large cohorts of microprolactinomas operated on via a transsphenoidal approach (at a tertiary center, by an experienced surgeon), the reported long-term remission rates were 72.1% and 74.3% (median follow-up ≥6 years) [14, 24]. The surgical complications rates were up to 4.7% and included permanent central hypothyroidism and permanent secondary adrenal insufficiency, as well as partial bitemporal hemianopsia. Other reported complications were postsurgical rhinorrhea, meningitis, and transient diabetes insipidus [14, 24]. Compared with surgical treatment, cabergoline treatment in men with microprolactinoma is prolonged (sometimes lifelong), but probably more effective in the long term, despite being given at low doses, usually once weekly. Importantly, the rate of side effects is low (4.3% in the current study), while the reported side effects are mild and usually disappear with dose reduction.

Our data raise another therapeutic aspect: cabergoline treatment in men with microprolactinomas is not only effective, but also has a rapid onset. The median time to prolactin normalization was 4.0 months, and the median time from prolactin normalization to achieving normal testosterone levels was 2.0 months. Obviously, these time estimates are prone to overestimation, because there is always a time lag between the patient’s prolactin and testosterone normalization and the next blood test that documents the hormonal improvement.

We report the results of cabergoline discontinuation in 13 men who already achieved prolactin control with treatment. In our cohort, 5 patients (38.5%) remained in remission and at the end of follow-up, with the only predictor of successful drug discontinuation was prolonged duration of cabergoline treatment: men who maintained normal prolactin levels were treated with cabergoline for a median of 10.0 years, compared with a median of 2.0 years in those with hyperprolactinemia relapse. All men who discontinued cabergoline treatment after prolactin suppression lasting more than 5 years achieved sustained remission. The rates of successful treatment discontinuation that we found are like those reported by Colao and colleagues in a large prospective study that also included 11 men with microprolactinomas, of whom 4 men (36.4%) achieved prolonged remission after cabergoline withdrawal [18]. A large meta-analysis of 743 patients with prolactinoma, by Dekkers and colleagues, also found that longer treatment duration was associated with higher rates of successful dopamine agonist discontinuation [19].

This is the first cohort study dedicated to investigating the outcomes of drug treatment in male patients with microprolactinoma. To the best of our knowledge, this is the largest cohort of cabergoline-treated men with microprolactinomas. However, microprolactinomas are rare in men, and the small available cohort size, reflected in a small number of outcomes, is a limitation. Similarly, due to the small population size, the lack of statistical significance does not necessarily prove a lack of association. The retrospective nature of our study is also a limitation. Despite our uniform treatment protocol and guidelines recommendation to escalate treatment doses in non-responders, some patients whose prolactin levels did not normalize were treated with a maximal cabergoline dose of 1.5 mg/week. It is possible that higher weekly doses would have normalized their prolactin levels, with good tolerability. Yet, other factors may influence treatment doses (such as patient preference, resolution of symptoms in the absence of prolactin normalization, or treatment side effects). In all cases, treatment discontinuation occurred after at least two years of low-dose cabergoline treatment. However, the decision to discontinue treatment and the timing of drug discontinuation were not determined according to a uniform protocol but rather at the discretion of the treating physician (based on imaging findings, patient wishes, and individual physician decision), indicating that this analysis is subject to selection bias. Additionally, patient-reported outcomes (e.g., sexual dysfunction) and patient-reported discontinuation of treatment might have been subject to reporting bias. Finally, the analysis comparing patient characteristics according to the outcome after cabergoline discontinuation (Table 4) does not have the statistical power required to conclude a lack of association between exposure and outcome, due to the small sample size.

In conclusion, we found that 89.4% of men with microprolactinoma achieved normal prolactin levels with cabergoline treatment. All 42 men that responded to cabergoline had normal testosterone levels at last clinic visit. We found that treatment with cabergoline took effect rather quickly: the median time to prolactin normalization was 4.0 months, and the median time from prolactin normalization to achieving normal testosterone was 2.0 months. However, 5 men (10.6%) did not achieve prolactin normalization, of whom 3 men (60.0%) remained with hypogonadism at the end of follow-up. The rate of side effects was low (4.3%), and the reported side effects were mild and disappeared with dose reduction.

Additionally, we investigated a sub-cohort of 13 men that achieved prolactin normalization and attempted drug discontinuation. However, only 5 patients experienced sustained normoprolactinemia. Men who received longer treatment with cabergoline (for >5 years) maintained normal prolactin levels after drug withdrawal. We believe that these findings shed light on the choice of cabergoline treatment in men with microprolactinoma, and will help patients make an informed decision between the options of medical and surgical treatment.

## Data Availability

The data that support the findings of this study are available from the corresponding author upon reasonable request.
